# Diversity of Astrovirus in Goats in Southwest China and Identification of Two Novel Caprine Astroviruses

**DOI:** 10.1128/spectrum.01218-22

**Published:** 2022-07-07

**Authors:** Jiayi Wang, Chenxia Xu, Mengting Zeng, Cheng Tang

**Affiliations:** a College of Animal & Veterinary Sciences, Southwest Minzu University, Chengdu, China; b Key Laboratory of Qinghai-Tibetan Plateau Animal Genetic Resource Reservation and Utilization Chengdu, China; Changchun Veterinary Research Institute

**Keywords:** astrovirus, goats, diversity, genotypes, recombination, genome

## Abstract

A total of 232 goat fecal samples (124 diarrheic and 108 nondiarrheic) collected from 12 farms in Southwest China were tested for astrovirus using RT-PCR. A total of 16.9% (21/124) of diarrheic and 20.4% (22/108) of nondiarrheic samples were astrovirus-positive, and no statistical difference was found in the detection rate between healthy and sick goats. Furthermore, 28 obtained complete ORF2 sequences could be classified into six genotypes according to the species classification criteria of the International Committee on Taxonomy of Viruses (ICTV). It is worth noting that, in addition to four known caprine astrovirus genotypes (*MAstV–33*, *MAstV–34*, *Caprine Astrovirus G5.1*, and *Caprine Astrovirus G3.1*), *MAstV–13* and *MAstV–24* genotypes were identified in goats. Interestingly, five of 19 ORF2 sequences in the *Caprine Astrovirus G3.1* genotype showed possible intragenotypic recombination events. Furthermore, nearly complete caprine astrovirus genomes of *MAstV–13* and *MAstV–24* genotypes were obtained. The genome of the SWUN/ECJK3/2021 strain shared the highest similarity (62.0% to 73.9%) with astrovirus in *MAstV–13*, and clustered in the so-called human-mink-ovine (HMO) clade, which contained the majority of the neurotropic astrovirus strains. Moreover, the SWUN/LJK2-2/2020 strain showed the highest similarity (69.7% to 78.6%) and the closest genetic relationship to the known porcine and bovine astroviruses in *MAstV–24*. In conclusion, this study confirmed six genotypes of astrovirus circulating among goats in Southwest China, including *MAstV–13* and *MAstV–24* genotypes. These findings enhance our knowledge of the prevalence and diversity of astroviruses.

**IMPORTANCE** Caprine astrovirus is a newly emerging virus, and information regarding its prevalence and molecular characteristics remains limited. In this study, six genotypes of astrovirus, including *MAstV–13* and *MAstV–24*, were identified in goats, adding two novel caprine astrovirus genotypes to the four previously known genotypes, thereby enriching the diversity of the caprine astrovirus. Moreover, genomes of *MAstV–13* SWUN/ECJK3/2021 and *MAstV–24* SWUN/LJK2-2/2020 strains were obtained from goats, which aids in the understanding of the infection spectrum and host range of the two genotypes. This study is the first to demonstrate the presence of neurotropic-like astrovirus (*MAstV–13*) in goats, which has significant implications for the diagnosis of neurological diseases in goats.

## INTRODUCTION

Astroviruses (AstVs) belong to the *Astroviridae* family and are divided into two genera, namely, *Mamastravirus* (*MAstV*) and *Avastrovirus* (*AAstV*). AstVs can infect a wide range of hosts and have been detected in more than 80 different mammalian and avian species (1). Specific AstV genotypes have been associated with gastrointestinal and neurological diseases (2–5), and asymptomatic manifestations thereof are also common (2, 3). According to the latest International Committee on the Taxonomy of Viruses (ICTV) proposal, the taxonomy of *MAstVs* depends on the original host species and the genetic distance of the complete capsid protein (>0.338) (6). Specifically, *MAstV* includes 19 recognized genotypes (*MAstV–1* to *19*), 16 proposed genotypes (*MAstV–20* to *35*), and several undefined genotypes (6–8).

The AstV genome comprises a 5′ untranslated region (5′UTR), three open reading frames (ORF1a, ORF1b, and ORF2), a 3′UTR, and a poly (A) tail (6). Nonstructural protein 1a and RNA-dependent RNA polymerase are encoded by ORF1a and ORF1b, respectively, while ORF2 encodes the viral capsid proteins (9). Furthermore, ORF2 is divided into two regions: a highly conserved region (1 to 415 amino acids (aa), N-terminus) and an extremely variable region (416aa-, C-terminus), which is the neutralizing antigenic determinant of the virus with multiple neutralization epitopes (10, 11). Recombination is a major evolutionary mechanism for AstVs that can rapidly generate novel divergent viruses (12, 13). In recent years, advances in next-generation sequencing (NGS) technologies have resulted in the discovery of multiple divergent AstV genotypes in various animals (2, 7), suggesting that some AstVs are capable of cross-species transmission and adaptation (14), further questioning the strict species specificity of AstV in livestock (15–17).

Caprine AstV was first discovered in Switzerland in 2019 by the means of NGS technologies, and three genotypes (*Caprine Astrovirus G5.1*, *Caprine Astrovirus G3.1*, and *MAstV–34*) were discovered (15). Subsequently, the *MAstV–33* genotype was identified in goats in China by our laboratory (18). The genomes of these four genotypes have been published in GenBank. However, due to the lack of appropriate cell culture systems for AstV isolation and limited epidemiological data, the association between caprine AstV and disease requires further investigation. Considering this, the current study aimed to investigate AstV diversity in goats, resulting in the identification of *MAstV–13* and *MAstV–24* in addition to the four known genotypes.

## RESULTS

### Detection of AstVs.

Fecal samples (*n* = 232) from 12 goat farms in three provinces were screened for AstV using RT-PCR. Approximately 18.5% (43/232) of the fecal samples were AstV-positive at a rate of 16.9% (21/124) in diarrheic and 20.4% (22/108) in nondiarrheic samples. Moreover, nine of the 12 farms were AstV-positive and AstVs were distributed in two of the three provinces (Table 1). The detection rates in Sichuan and Chongqing provinces were 11.8% (18/152) and 41.7% (25/60), respectively.

### Identification of six genotypes of AstVs in goats.

Among 43 AstV-positive samples, 28 complete ORF2 sequences were successfully obtained (GenBank accession no. OK107512-OK107515, OM890909-OM890932, ON571622), and six distinct genotypes (*MAstV–33*, *MAstV–34*, *Caprine Astrovirus G5.1*, *Caprine Astrovirus G3.1*, *MAstV–13* and *MAstV–24*) were identified in terms of capsid protein genetic distances. Notably, in addition to the four known caprine AstV genotypes (*MAstV–33*, *MAstV–34*, *Caprine Astrovirus G5.1*, and *Caprine Astrovirus G3.1*) (15, 18), *MAstV–13* and *MAstV–24* were identified in goats. Interestingly, distinct genotypes of AstVs were simultaneously found in four of nine AstV-positive farms (Table 1). In addition, coinfection with two AstV strains was found in individual fecal samples from three goats (LJK2, LJK3, and LFX2), in which LJK2 and LJK3 were coinfected with *Caprine Astrovirus G3.1* and *MAstV–24*, while LFX2 was coinfected with *Caprine Astrovirus G3.1* and *Caprine Astrovirus G5.1*.

**TABLE 1:** Details of sample information and RT-PCR detection of caprine AstV

Province	Sampling location	Genotypes	Total no. of samples	No. of positive samples	Positive rate (%)
Sichuan	Sichuan I		20	0	0.0
	Sichuan II		20	0	0.0
	Sichuan III	*Caprine Astrovirus G3.1*	12	4	33.3
		*Caprine Astrovirus G5.1*			
		*MAstV–24*			
	Sichuan IV	*Caprine Astrovirus G3.1*	45	2	4.4
		*MAstV–33*			
	Sichuan V	*Caprine Astrovirus G3.1*	35	5	14.3
		*MAstV–33*			
		*MAstV–34*			
	Sichuan VI	*Caprine Astrovirus G3.1*	20	7	35.0
		*MAstV–33*			
		*MAstV–34*			
Chongqing	ChongqingI	*Caprine Astrovirus G3.1*	12	6	50.0
	Chongqing II	*Caprine Astrovirus G3.1*	12	5	41.7
	Chongqing III	*Caprine Astrovirus G3.1*	12	7	58.3
	Chongqing IV	*MAstV–13*	12	5	41.7
	Chongqing V	*MAstV–13*	12	2	16.7
Yunnan	Yunnan I		20	0	0.0
Total			232	43	18.5

The positive rates of six AstV genotypes are shown in Table 2. Moreover, *Caprine Astrovirus G3.1* was the predominant genotype (67.9%), which was distributed in seven of the nine AstV-positive farms.

**TABLE 2:** Proportion of six genotypes from 28 obtained ORF2 sequences

Genotypes	Positive samples	Positive rate (%)
*Caprine Astrovirus G3.1*	19	67.9
*MAstV–13*	2	7.1
*MAstV–24*	2	7.1
*MAstV–33*	2	7.1
*MAstV–34*	2	7.1
*Caprine Astrovirus G5.1*	1	3.6

### Complete ORF2 gene sequencing and phylogenetic analysis.

Among the four known genotypes, the lengths of 19 ORF2 sequences in *Caprine Astrovirus G3.1* ranged from 2,244 to 2,259 bp, encoding 747 to 752 aa. They shared 81.5% to 100.0% aa similarity with each other and 82.9% to 93.3% aa similarity with the only *Caprine Astrovirus G3.1* strain in GenBank (CapAstV-G3.1). The two *MAstV–33* ORF2 sequences were 2,313 bp long and encoded 770 aa. They shared 99.0% aa similarity with each other and 65.9% to 80.1% aa similarity with all known complete *MAstV–33* ORF2 sequences available in GenBank (Bovine AstV (BAstV/BoAstV)-B76-2/HK, BAstV-B18/HK, BoAstV/JPN/Kagoshima1-7/2014, BufAstGX-M522, BufAstGX-M541, BufAstV-NND-s2, BAstV-GX7, BAstV-GX27, BAstVGX-G1, BAstGX-J7, BAstGX-J8, BAstGX-J22, BAstGX-J27, BoAstv/CHN/Hunan-1/2019, BoAstv/CHN/HLJ-2/2019, CcAstV-1/DNK/2010, CcAstV-2/DNK/2010, CcAstV/roe deer/SLO/D12-14/2014, CcAstV/roe deer/SLO/D5-14/2014, Sichuan takin astrovirus, CapAstV-SWUN/F4/2019). The two *MAstV–34* ORF2 sequences were 2,193 and 2,202 bp long and encoded 730 and 733 aa, respectively. They shared 79.3% aa similarity with each other and 66.7% to 79.4% aa similarity with the complete known ORF2 sequences of *MAstV–34* (BoAstV/JPN/Kagoshima2-3-2/2015, BoAstV/JPN/Hokkaido11-55/2009, CapAstV-G2.1, BoAstv/CHN/Henan-1/2019). The *Caprine Astrovirus G5.1* sequence was 2,256 bp long, encoded 751 aa, and shared 73.2% to 78.0% aa similarity with the complete known ORF2 sequences of *Caprine Astrovirus G5.1* (CapAstV-G5.1, OvAstV-S5.1, OvAstV-S6.1).

Interestingly, the two *MAstV–24* strains were 2,259 bp long and encoded 752 aa. They shared 100.0% aa similarity with each other and showed the highest aa similarity (98.5%) with the ovine AstV-2 strain (OvAstV-2, GenBank accession no. JN592482), which was found in a healthy domestic sheep in Hungary (19). Additionally, the *MAstV–24* strains shared 73.2% to 82.2% aa similarity with *MAstV–24* strains from other hosts. Notably, two *MAstV–13* strains were 2,280 bp long and encoded 759 aa. They shared 100.0% aa similarity with each other and shared the highest aa similarity (76.0%) with the enterotropic Ovine AstV-1 (OAstV-1, NC_002469) (20), which is closely related to neurotropic strains. Moreover, the *MAstV–13* strains shared 65.1% to 72.7% aa similarity with other known neurotropic AstV strains from ruminants.

**FIG 1:**
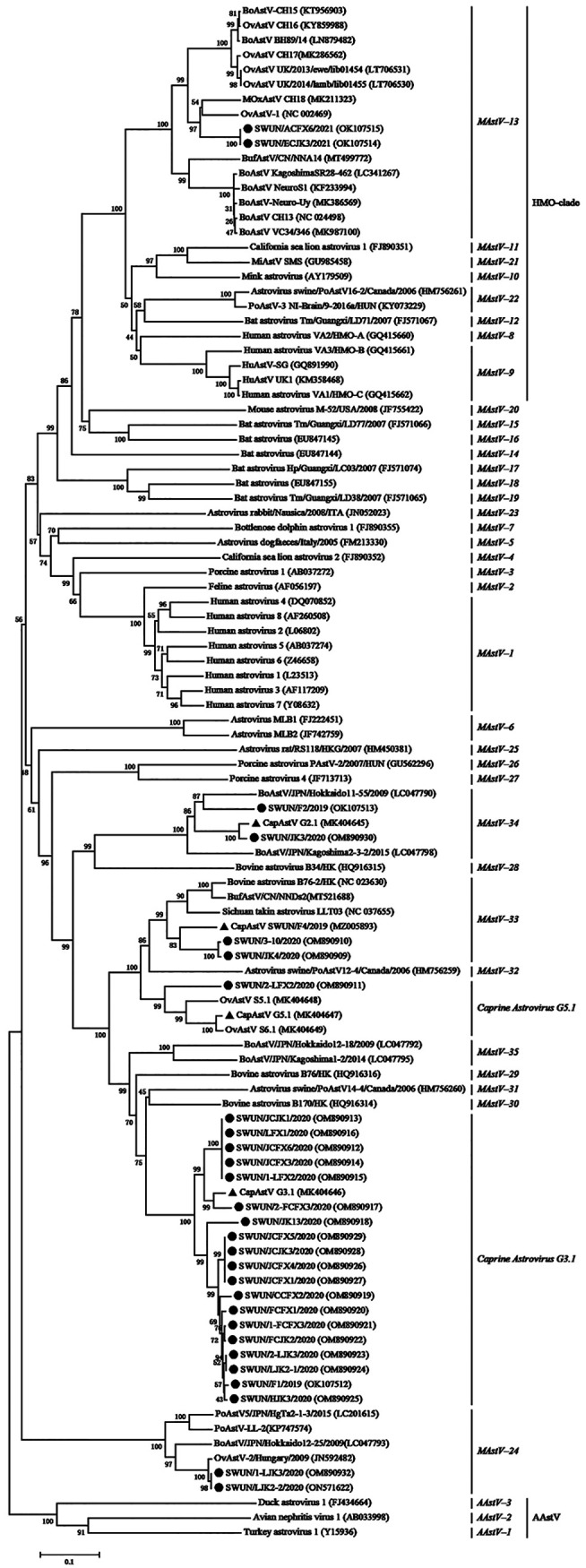
Phylogenetic tree based on complete amino acid sequences of ORF2 gene. Sequence alignments and clustering were performed using ClustalW in MEGA 7.0 software. The tree was constructed using the neighbor-joining method with bootstrap values calculated for 1,000 replicates. Twenty-eight caprine astrovirus strains from this study were marked with black circles, and four known caprine AstV strains were marked with black triangles.

A neighbor-joining phylogenetic tree of the complete ORF2 aa sequences was constructed based on the reference strains from *MAstV–1* to *MAstV–35* as well as representative strains from GenBank with high similarity to our strains (Fig. 1). The resulting tree revealed that 28 AstV strains in this study could be divided into six distinct branches. Two (3-10 and JK4) of the 28 strains belonged to *MAstV–33* and formed a subgroup that was closely related to BufAstV/CN/NNDs2 (GenBank accession no. MT521688), BAstV-B76-2/HK (GenBank accession no. NC_023630), Sichuan takin AstV (GenBank accession no. NC_037655) and CapAstV-SWUN/F4/2019 (GenBank accession no. MZ005893). One (2-LFX2) of the 28 strains belonged to *Caprine Astrovirus G5.1* and had the closest relationship with OvAstV-S5.1 (GenBank accession no. MK404648), OvAstV-S6.1 (GenBank accession no. MK404649) and CapAstV-G5.1 (GenBank accession no. MK404647), all of which were found in sheep/goats in Switzerland. Interestingly, 19 of the 28 strains belonged to *Caprine Astrovirus G3.1*, were clustered together with the Swiss caprine AstV strain CapAstV-G3.1 (GenBank accession no. MK404646), and could be further divided into three distinct subclades. Two (JK3 and F2) of the 28 strains belonged to *MAstV–34* and were clustered with CapAstV-G2.1 (GenBank accession no. MK404645), found in Swiss goats, and BoAstV Hokkaido11-55 (GenBank accession no. LC047790), found in Japanese cattle, respectively. Notably, two strains (LJK2-2 and 1-LJK3) were phylogenetically separated from known caprine AstV strains and clustered with OvAstV-2 strain found in sheep in Hungary. This cluster has been reported as *MAstV–24* and has been identified yet in sheep, pigs, and cattle (19, 21–26). In contrast, ACFX6 and ECJK3 strains were clustered in *MAstV–13*, which contained the major neurotropic AstV strains from ruminants (5). These results suggest that AstVs have a broader spectrum of host species and a higher genetic diversity than previously estimated.

### Recombination analysis of ORF2 sequences.

**FIG 2:**
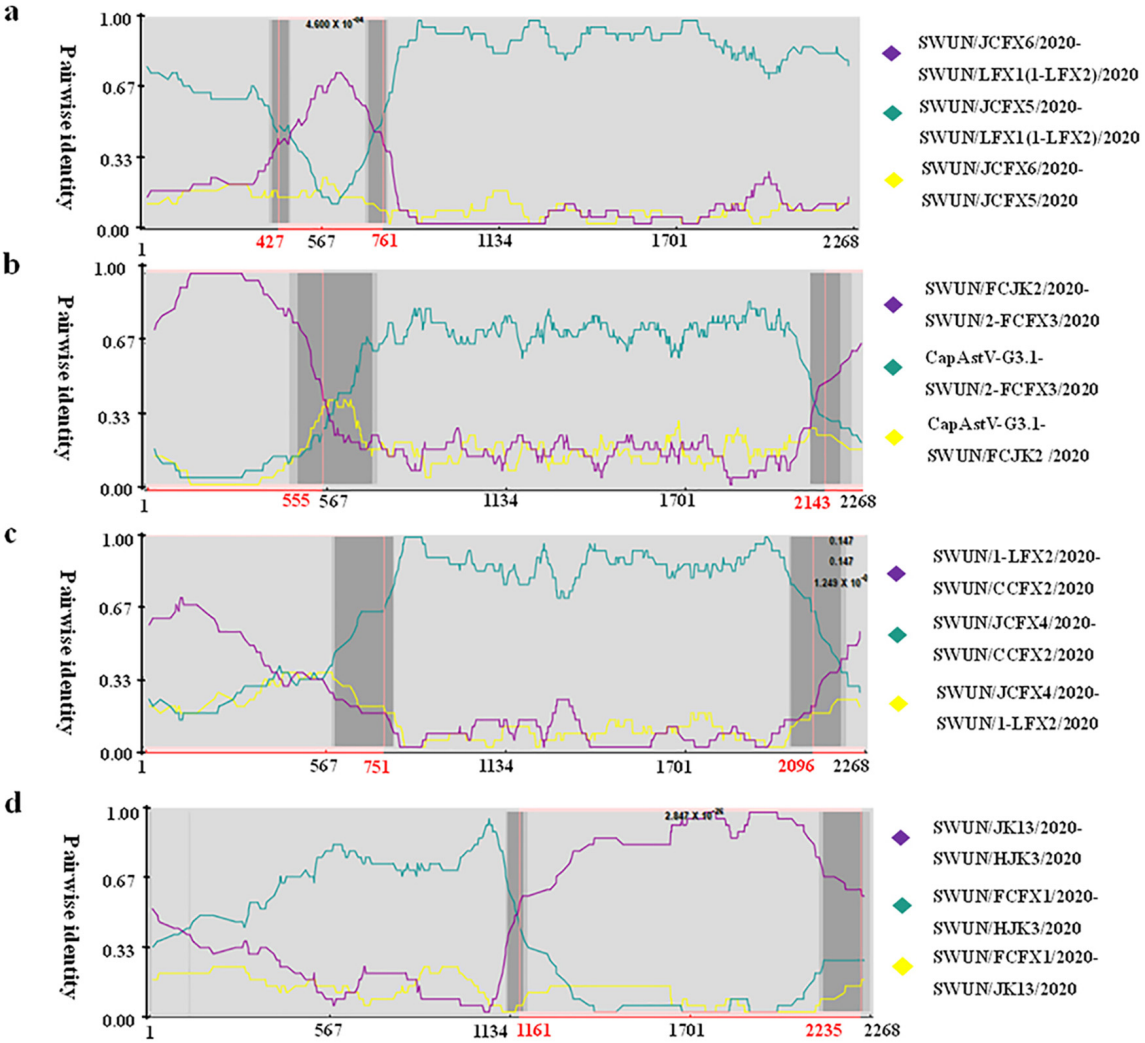
Recombination analysis of newly discovered complete ORF2 genes of caprine astrovirus. (a) Predicted recombination events 1 and 2 with SWUN/LFX1/2020 and SWUN/1-LFX2/2020 as recombinants, respectively, SWUN/JCFX6/2020 as the major parent and SWUN/JCFX5/2020 as the minor parent. (b) Predicted recombination event 3 with SWUN/2-FCFX3/2020 as the recombinant, CapAstV-G3.1 as the major parent and SWUN/FCJK2/2020 as the minor parent. (c) Predicted recombination event 4 with SWUN/CCFX2/2020 as recombinant, SWUN/JCFX4/2020 as the major parent and SWUN/1-LFX2/2020 as the minor parent. (d) Predicted recombination event 5 with SWUN/HJK3/2020 as recombinant, SWUN/FCFX1/2020 as the major parent and SWUN/JK13/2020 as the minor parent. Plots are constructed using the RDP Method graphical output in RDP4. Nucleotide positions within the ORF2 genes of astrovirus are depicted on the axis of abscissas in kb. Red bars schematically indicate the parts of the ORF2 genes involved in the recombination events.

Recombination analysis was performed on the 28 ORF2 sequences of caprine AstV; however, recombination events were only predicted in *Caprine Astrovirus G3.1*. Five (LFX1, 1-LFX2, 2-FCFX3, CCFX2, and HJK3) of 19 *Caprine Astrovirus G3.1* strains were identified as recombinants by at least five methods using Recombination Detection Program 4.0 (RDP 4.0, version 4.96), and their putative parental strains belonged to *Caprine Astrovirus G3.1*. All these events were predicted to be regional recombination events occurring in different regions of ORF2, as shown in Fig. 2a to d. To our knowledge, this is the first report of recombination events in the *Caprine Astrovirus G3.1* genotype.

Two of the five recombination events were similar; both took JCFX6 as the major and JCFX5 as the minor parent, both of which were from the same farm in Chongqing municipality. Moreover, both recombination breakpoints were predicted at nucleotide (nt) positions 427 and 761 in LFX1 (recombination event 1) (Fig. 2a) and 1-LFX2 (recombination event 2) (Fig. 2a). Recombination event 3 was predicted in strain 2-FCFX3, and the putative major and minor parental strains were CapAstV-G3.1 and FCJK2, respectively, with the breakpoint at nt positions 555 and 2,143, respectively (Fig. 2b). Two recombination breakpoints associated with the CCFX2 recombinant (recombination event 4) were predicted at nt positions 751 and 2,096, with JCFX4 and 1-LFX2 as the putative major and minor parental strains, respectively (Fig. 2c). In addition, recombination event 5 was identified in the HJK3 strain, and its putative parental strains were established as FCFX1 and JK13, with breakpoints at nt positions 1,161 and 2,235, respectively (Fig. 2d).

### Genomic characterization of *MAstV–13* SWUN/ECJK3/2021 strain in goats.

**FIG 3:**
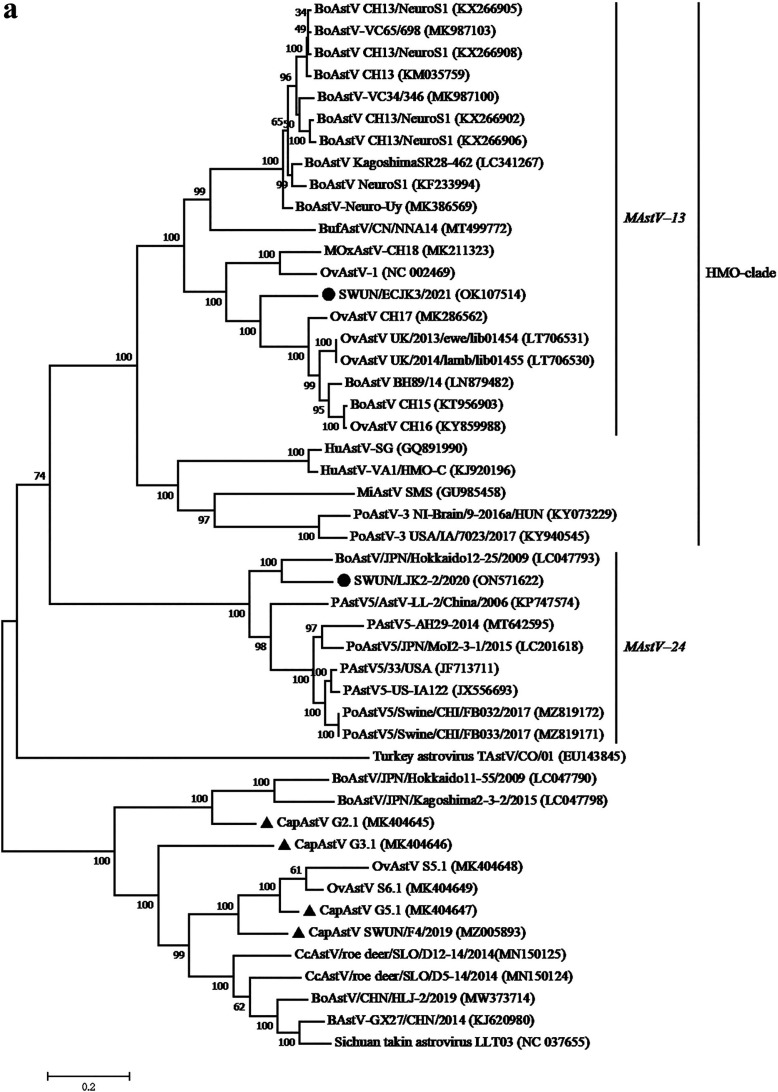

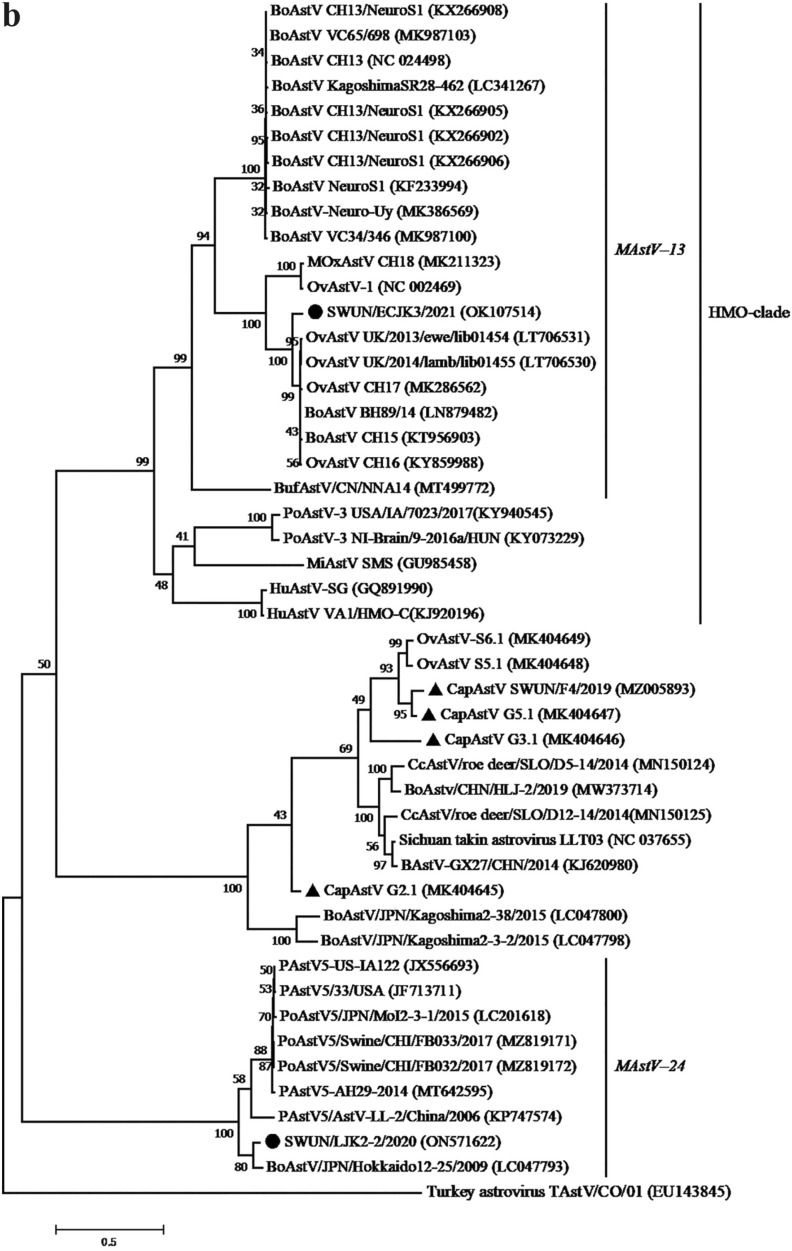

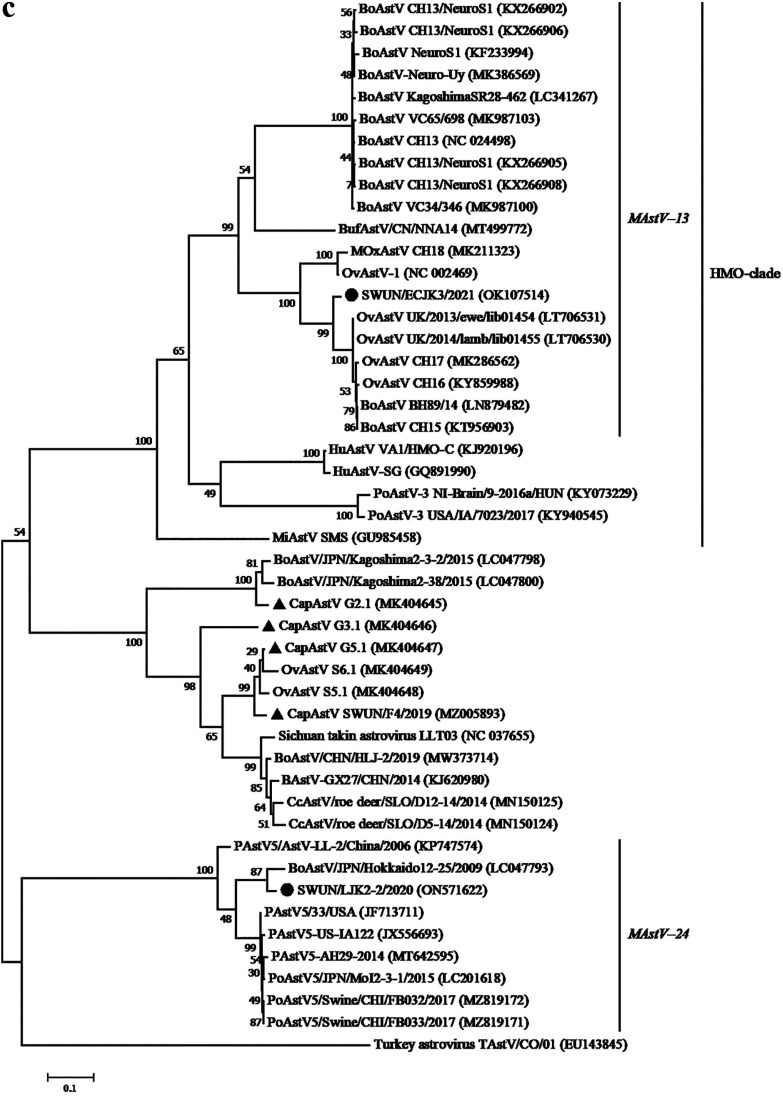
Phylogenetic trees based on nucleotide sequences of genome (a), complete amino acid sequences of ORF1a (b) and ORF1b (c). Sequence alignments and clustering were performed by ClustalW in MEGA 7.0 software. The trees were constructed using the maximum likelihood method with bootstrap values calculated for 1,000 replicates. Two strains from this study were marked with black circles, and four known caprine astrovirus strains were marked with black triangles.

A nearly complete AstV genome of 6,387 nt in length, which contained three complete putative overlapping ORFs, was successfully obtained and named SWUN/ECJK3/2021 (GenBank accession no. OK107514). ORF1a and ORF1b were 2,550 nt (849 aa) and 1,566 nt (521 aa) in length, respectively, with a 46 nt overlap. The ribosomal frameshift signal sequence (5′-AAAAAAC-3′) was identified near the ORF1a 3′ end, resulting in a polyprotein (ORF1ab) of 1,356 aa. ORF2 was 2,280 nt (759 aa) long and overlapped with ORF1b by 8 nt. The genome of SWUN/ECJK3/2021 shared the highest nt similarity (73.9%) with the neurotropic OvAstV UK/2013/ewe/lib01454 strain, which was found in a mature domestic sheep with neurological disease (27). Additionally, ORF1ab displayed the highest aa similarity (92.0%) to OvAstV-UK/2014/lamb/lib01455, while ORF2 shared the highest aa similarity (76.0%) with OAstV-1 (20). Phylogenetic analysis based on the genome, ORF1a, and ORF1b confirmed that the SWUN/ECJK3/2021 strain and six neurotropic AstVs of the BoAstV-CH15/OvAstV-CH16 group (BoAstV-BH89/14 and -CH15, OvAstV-UK/2013/ewe/lib01454, -UK/2014/lamb/lib01455, -CH16, and -CH17) were clustered into a monophyletic branch, and SWUN/ECJK3/2021 was clustered into a small independent branch (Fig. 3a to c). The ORF2 phylogenetic tree has been previously described (Fig. 1).

### Genomic characterization of *MAstV–24* SWUN/LJK2-2/2020 strain in goats.

A nearly complete AstV genome with a size of 6,232 nt, containing three complete putative overlapping ORFs, was successfully obtained and named SWUN/LJK2-2/2020 (GenBank accession no. ON571622). ORF1a (2,526 nt) and ORF2 (2,214 nt) encoded proteins of 841 and 737 aa, respectively. The translation of ORF1b (1,515 nt) may be linked to ORF1a by a ribosomal frame-shifting mechanism, resulting in an ORF1ab of 1,332 aa. The genome of the SWUN/LJK2-2/2020 strain shared the highest nt similarity (78.6%) with BoAstV/JPN/Hokkaido12-25/2009, which was the only BoAstV in *MAstV–24* found in the feces of a Japanese diarrheal calf (21). Additionally, ORF1ab also showed the highest similarity to BoAstV/JPN/Hokkaido12-25/2009 (93.7%), whereas ORF2 shared the highest aa similarity with the OAstV-2 strain (98.5%), for which the full genomic sequence was unavailable. The phylogenetic tree of the genome, ORF1a, and ORF1b, revealed that the closest relative of SWUN/LJK2-2/2020 was BoAstV Hokkaido12-25/2009 (Fig. 3a to c). Additionally, the ORF2 phylogenetic tree has been previously described (Fig. 1).

## DISCUSSION

### Diversity of AstVs in goats in Southwest China.

Being a newly emerging virus (15, 18), knowledge of the epidemiology and molecular characteristics of the caprine AstV remains limited. Here, 18.5% (43/232) of goat fecal samples were detected as AstV-positive using RT-PCR, and the virus was detected in nine of the 12 farms located across two provinces (Sichuan and Chongqing), suggesting that caprine AstVs circulated among goats in Southwest China. Because the associated fecal samples in Yunnan province were collected from a single farm, the results may be biased due to the limited sampling site. Furthermore, no statistical difference was found in the detection rate of AstV between the diarrheic (16.9%) and nondiarrheic (20.4%) samples. Because the detection assay in this study targeted the 3′ end of the viral ORF1b, which could not determine the genotype of AstVs, the association between specific caprine AstV genotypes and disease warrants further investigation. In fact, disease association in mammalian species is less evident, with enteric AstVs infections being mainly asymptomatic (7, 28).

Among the 43 AstV-positive samples, 28 complete ORF2 sequences were successfully obtained and could be divided into six genotypes according to the ICTV species classification criteria (6). Interestingly, in addition to four known caprine AstV genotypes (*MAstV–33*, *MAstV–34*, *Caprine Astrovirus G5.1*, and *Caprine Astrovirus G3.1*) (15, 18), *MAstV–13* and *MAstV–24* were identified in goats. These results underscored the remarkable diversity of AstVs harbored by goats. Caprine AstVs now appear in six distinct clades of the evolutionary tree that involve many other mammalian species, suggesting different ancestral origins and potential interspecies transmissions (5, 15, 19, 21). Interestingly, distinct genotypes of AstVs could be found simultaneously on the same farm (Table 1), indicating that one farm could serve as a reservoir for different caprine AstV strains (29). Moreover, individual goats were coinfected with different AstV genotypes, which is similar to previously reported cases in cattle (17) and pigs (24). The occurrence of coinfection within the same host is of great significance for the evolution of AstVs given that it is an essential step for recombination to occur (13).

### The recombination events of ORF2 gene in the *Caprine Astrovirus G3.1* genotype.

Recombination events of AstVs inter- or intragenotypic have been increasingly reported in recent years, which is the major mechanism contributing to the emergence of novel AstV lineages (7, 12, 13, 30). In this study, intragenotypic recombination events were predicted in five of 19 strains in the *Caprine Astrovirus G3.1* genotype (Fig. 2a to d), which was in accordance with previous reports on the intragenotypic recombination between BoAstVs (8) and between porcine AstVs (PoAstVs) (31). Interestingly, five recombination events occurred in different regions of ORF2, which can diversify viral capsid proteins, thus affecting the antigenicity of the virus and enabling it to escape host immunity (8, 32). In fact, ORF2 sequences, such as FCFX1 and JK13 in *Caprine Astrovirus G3.1*, also showed strong recombinant signals, but only the major or minor parental strain could be identified due to limited AstV sequence availability. Together with the recombination events identified in this study, recombination events have been predicted in four of six known genotypes of caprine AstVs, including *Caprine Astrovirus G5.1* (CapAstV-G5.1), *Caprine Astrovirus G3.1* (LFX1, 1-LFX2, 2-FCFX3, CCFX2 and HJK3), *MAstV–33* (CapAstV-SWUN/F4/2019), and *MAstV–34* (CapAstV-G2.1) (15, 18). These events suggest that recombination plays an essential role in the evolution of caprine AstV. Recognition of the genetic diversity and evolution of AstVs among animal populations is of great significance to fully understand the virus (13).

### The genomic characterization of a neurotropic-like AstV (*MAstV–13*) in goats.

From 2010 onwards, specific AstV genotypes were found to be associated with neurological diseases in humans, minks, cattle, pigs, sheep, muskox, and alpaca (5, 27, 33–38). Strikingly, the majority of neurotropic AstVs were genetically closely related and clustered together in the so-called human-mink-ovine (HMO) clade, while most enteric AstVs are much more diverse (4, 7). In addition to the central nervous system, neurotropic AstVs can be detected in feces or nasal swabs, indicating that the gastrointestinal and respiratory tracts may serve as viral reservoirs (4, 5, 16). In this study, phylogenetic analysis clustered two ORF2 strains (SWUN/ECJK3/2021 and SWUN/ACFX6/2021) into the *MAstV–13* genotype, which included the majority of the neurotropic AstVs from ruminants (5). Moreover, the SWUN/ECJK3/2021 genome demonstrated the highest similarity (73.3% to 73.9%) and closest genetic relationship with neurotropic AstVs of the BoAstV-15/OvAstV-CH16 group, which contains several bovine and ovine neurotropic AstV strains (27, 39–42), suggesting a similar genetic ancestry or common genetic features. Regrettably, since the goats sampled in this study did not exhibit neurological symptoms, no nervous tissue samples were collected; thus, we named the strain neurotropic-like AstV.

Indeed, detecting neurotropic AstVs in ruminant fecal samples may be expected. In 2020, Kauer et al. found neurotropic AstVs (BoAstV CH13/NeuroS1) in calf feces (16), and in 2021, Fang et al. reported a neurotropic-like AstV in the feces of water buffalo (43). These events indicate that fecal shedding may constitute a route of neurotropic AstV transmission (16), for which additional supporting data are presented in this study. Our results demonstrated the presence of neurotropic-like AstVs in goats, which has implications for the diagnosis of neurological diseases in goats in the future.

### Genomic characterization of the *MAstV–24* SWUN/LJK2-2/2020 strain.

A *MAstV–24* genotype strain (SWUN/LJK2-2/2020) was identified in goats in the present study. Two characteristics were observed in this novel strain: (i) SWUN/LJK2-2/2020 was closely related to the Japanese BoAstV Hokkaido12-25 strain, not only in the capsid protein (83.0%), but also in the predicted nonstructural proteins 1ab (92.8%), and genome nucleotides (78.6%). Additionally, it shared high capsid protein similarity with OvAstV-2 (98.5%), for which the full genomic sequence is unavailable (19), raising the question of transmission events between sheep, cattle, and goats (19, 21); and (ii) numerous aa mutations were found in the capsid protein of SWUN/LJK2-2/2020 compared with BoAstV and known PoAstV type 5 strains in *MAstV–24* (21–26). Thus, it could be inferred that the sequence of AstV has changed in the process of adaptation to goats, resulting in AstV heterogeneity within the same genotype.

Although the source of *MAstV–24* caprine strains could not be identified, it can be speculated that the high density of farm animals provides an environment that favors cross-species transmission of AstV between domestic animals (2). AstV was once considered a species-specific virus; however, the discovery of multiple novel AstVs and recent genetic studies have provided evidence that specific AstV genotypes may have the potential to cross species barriers and infect a broader spectrum of hosts (7, 9, 14).

### Conclusions.

In this study, six genotypes of AstVs circulating among goats unveiled the diversity of AstVs in goat hosts. Moreover, in addition to four known caprine AstV genotypes (*MAstV–33*, *MAstV–34*, *Caprine Astrovirus G5.1*, and *Caprine Astrovirus G3.1*), *MAstV–13* and *MAstV–24* genotypes were identified in goats, and their genomes were successfully obtained. Our findings contribute to a better understanding of the genetic diversity and evolution of caprine AstV.

## MATERIALS AND METHODS

### Samples collection.

A total of 232 (124 diarrheic and 108 nondiarrheic) goat fecal samples were collected from 12 farms in southwest China from December 2019 to December 2021. These farms located in Sichuan province, Chongqing municipality and Yunnan province, and the number of the collected samples ranged from 12 to 45 animals per farm (Table 1). The goats on the farm were less than 2 months old, and the diarrheic goats shared an abnormally frequent discharge of watery feces. All samples were shipped on ice and stored at −80°C in sterile 50-mL centrifuge tubes.

### Viral nucleic acid extraction.

Fecal samples were fully dispersed as 10% suspensions in phosphate-buffered saline (PBS) and centrifuged for eight min at 10,000 × *g* and 4°C, followed by filtration through a 0.22-μm filter. Then, 400 μL of each fecal supernatant was used for viral RNA extraction, using RNAios Plus (TaKaRa Bio Inc., Shiga, Japan) according to the manufacturer's instructions.

### Screening for caprine AstV by RT-PCR.

In this study, an RT-PCR assay for detecting caprine AstV was established after analyzing the genomes of four known caprine AstV genotypes, the specificity and reproducibility has been validated and the detection limit is 2.49 × 10^3^ copies·μL^−1^. Briefly, a pair of primers (CapAstV 300-Forward: 5′-TTTGGBATGTGGGTTAARCCwG -3′, CapAstV 300-Reverse: 5′-TTGGTCCKCCCCTCCAAAGA -3′) was used to amplify a 300-bp fragment of the 3′ conserved region of AstVs ORF1b gene (position 3,581 to 3,908 bp of the CapAstV-G5.1 genomic sequence, GenBank accession no. MK404647). The first-strand cDNA was synthesized using the PrimeScript RT reagent kit (TaKaRa Bio, Inc.) according to the manufacturer’s instructions. The PCR was set up in 25-μL reaction volume containing 12.5 μL of EmeraldAmp PCR Master Mix (2×Premix) (TaKaRa Biotechnology Co., Ltd., People’s Republic of China), 0.05 μM forward primer, 0.05 μM reverse primer, 2 μL of cDNA and an appropriate volume of double-distilled water. The mixtures were carried out with the following setting: 4 min, 94°C, 35 cycles each of 30 s, 94°C; 30 s, 57.5°C; 30 s, 72°C, and final extension 10 min, 72°C. The 300-bp amplification products were sequenced using Sanger sequencing (3730xl, ABI, USA). A significance test of the detection rates of AstV in diarrheic and nondiarrheic fecal samples was performed using SPSS software.

### Complete ORF2 gene sequence amplification.

To determine the genotype of AstV-positive samples, a series of primers were designed based on the genomes available in GenBank to amplify the full-length ORF2 sequences of each genotype (see Table S1). All amplification products were cloned into the vector pMD18-T (cat. no. 6011, TaKaRa, Dalian, China) and confirmed by sequencing.

### The genome amplification of *MAstV–13* SWUN/ECJK3/2021 and *MAstV–24* SWUN/LJK2-2/2020 strains.

Two sets of primers were designed to amplify the genomes of *MAstV–13* and *MAstV–24* genotypes, respectively (Table S2), based on the conserved regions of AstV genomes (GenBank accession no. KT956903, LN879482, LT706531, LT706530, KY859988, MK286562, JN592482, and LC047793). The PCR products were purified and cloned into the pMD19-T simple vector prior to sequencing. The sequences were assembled using SeqMan software (version 7.0; DNASTAR Inc., WI, USA). Putative ORFs and their corresponding aa were predicted using the ORF Finder tool (https://www.ncbi.nlm.nih.gov/orffinder/).

### Sequences, phylogenetic, and recombination analysis.

Sequence similarity analyses were performed using the MegAlign program of DNASTAR 7.0 software (DNASTAR Inc.). MEGA v.7.0.21 was used to perform a multiple sequence alignment and the aa *p*-distance was calculated using the *p*-distance model. Phylogenetic analysis of the genomes, ORF1a, ORF1b, and ORF2 genes were also performed using MEGA v.7.0.21. Branch statistics were calculated by bootstrap analysis of 1,000 replicates. The accession numbers of the nucleotide sequences obtained in this study and reference sequences were shown in Table S3. Recombination analysis was performed using RDP 4.0 (version 4.96) with the RDP, GeneConv, Chimaera, MaxChi, BootScan, SiScan, and 3Seq methods. Briefly, the sequences with significant recombination event (*P* < 0.05) derived from at least five methods were presented.

### Data availability.

The sequences of AstV described in this study has been deposited in GenBank under accession numbers OK107512 to OK107515, OM890909 to OM890932, ON571622.
