# Supplementary material for: Diversity of Astrovirus in Goats in Southwest China and Identification of Two Novel Caprine Astroviruses
Source: Microbiol Spectr. 2022 Jul 7;10(4):e01218-22. doi: 10.1128/spectrum.01218-22 (PMC9430535; doi:10.1128/spectrum.01218-22)
Supplement: Supplemental file 1 — Supplemental material. Download spectrum.01218-22-s0001.pdf, PDF file, 0.2 MB [file spectrum.01218-22-s0001.pdf]

Table S1 Primer sequences for amplification of different genotypes of caprine AstV ORF2

Genotypes	Primer name (amplification)	Primer Sequences (5'-3')	Amplification size (bp)	Reference strains
MAstV–33	MAstV–33-F1	ATGGCTAGCAACAACACCC	1181	China/SWUN/F4/2019 (MZ005893)
	MAstV–33-R1	CCAATTCCTGTGTTACCAGG		
	MAstV–33-F2	TGGCTGGTGGTTGATCAAG	548	
	MAstV–33-R2	CATACTGAGCAGCCACAGCAT		
	MAstV–33-F3	ATAATGGTATAGGTTTCATGAT	966	
	MAstV–33-R3	CTAAACGCCCATAGCATA		
Caprine Astrovirus G5.1	G5.1-F1	ATGGCTAGCAACAACACAC	1004	Caprine astrovirus G5.1 (MK404647)
	G5.1-R1	AGCCACCAGCCACCCCTGAA		
	G5.1-F2	AATATGGCTTGTCACTGATAC	732	
	G5.1-R2	GGATGATCTGAGCCTGTGC		
	G5.1-F3	GGGCATACCAGTTTATCTGG	842	
	G5.1-R3	CTAGATCCCCAAGGCATG		
Caprine Astrovirus G3.1	G3.1-F1	CTTTGGAGGGGAGGACCAAA	1273	Caprine astrovirus G3.1 (MK404646)
	G3.1-R1	GACGCTCYACAGTGGTRGGTGT		
	G3.1-F2	TGCTAARATAACAACWGATG	1318	
	G3.1-R2	GATTAACTGCCAACCGAGCG		
	G3.1-F3	TGGCGYGTKGACAACTTY	743	
	G3.1-R3	ACTACACRGAAAACAGAAAA		
	G3.1-F1'	CTTTGGAGGGGAGGACCAAA	1129	Caprine astrovirus G3.1 (MK404646)
	G3.1-R1'	GGCWATGCACGGKACATC		
	G3.1-F2'	TGCAACCATAACMACYGAYG	1318	
	G3.1-R2'	GATTAACTGCCAACCGAGCG		
	G3.1-F3'	TGGCGYGTKGACAACTTY	743	
	G3.1-R3'	ACTACACRGAAAACAGAAAA		
MAstV–34	MAstV–34-F1	ATGCCACGTAATCGCAATGG	1115	Caprine astrovirus G2.1 (MK404645)
	MAstV–34-R1	GGTTTATTGTTCTGTGCATC		
	MAstV–34-F2	CCCTGGTATGCTCAATATGGT	931	
	MAstV–34-R2	CCTGCATCAACTTTCAACCT		
	MAstV–34-F3	CTGGTGCTAACACCCCAA	740	
	MAstV–34-R3	CACAAATGTCAGAAAAAGA		
MAstV–24	MAstV–24-F1	ATGGCCAATCAGCGTAAT	734	OAstV-2/Hungary/2009 (JN592482)
	MAstV–24-R1	AGCCAAAGTTCACCAGACCA		
	MAstV–24-F2	TGCTGGGCCAACCATAGAG	869	
	MAstV–24-R2	GAGCATCAACACCCATGGG		
	MAstV–24-F3	CAGCCTCATCTGTCTTTGG	911	
	MAstV–24-R3	TCACTCGGACTCACTCTCGT		
MAstV–13	MAstV–13-F1	ATGGCTGAAAAGCCCCAG	850	OvAstV-1_(NC_002469)
	MAstV–13-R1	TGATCTGCCCCTCAGTGTC		
	MAstV–13-F2	GGTGGGTTACTGACACCA	1527	
	MAstV–13-R2	CTAACGTGTACCACCCGT		
	MAstV–13-F3	TGCAGACATCCCAGAGGT	860	
	MAstV–13-R3	CCTGTACCCTCGATCCTACTC		

Table S2. PCR primers used for genome amplification and verification of SWUN/ECJK3/2021 and SWUN/LJK2-2/2020 strains

Strain (*genotype*)	Name	Primer sequence[a] (5′-3′)	Amplification size (bp)	Name	Primer sequence[b] (5′-3′)	Amplification size (bp)
ECJK3(*MAstV–13*)	1	GTAGGTCAGCCTTTGTAGTTC<br>GCCCTAAAGCAAGTACCR	1380	1	GTAGGTCGGCCTTTGTAGTT<br>GCCAGTACCCTCCTTTGA	1347
	2	AGCTTACTAATGGGCCTG<br>TTTGACTGGCTCTAGTTCT	1728	2	GTTCGCACTGCCGATGGC<br>GTGACACTAGTGGAGACGA	1211
	3	GAGTGGTCAGACCCTGGC<br>GGTATTGTGCCGTCATAGCG	1198	3	ACGTATTATGGCTGATGC<br>CGTGTGCGCCGTTTCATGCG	1151
	4	AAAACATCAAAGGTGCGTGA<br>ACATTAACCCTAAACCTA	1239	4	GAGCCCGACCAAACGTGC<br>CAATAGTAGCAGACACAG	1300
	5	ATGGCTGAAAAGCCCCAG<br>TGATCTGCCCCTCAGTGTC	850	5	TGCTGGATAAGCTTTGGAG<br>CAAATAGGGACAGCTGCT	1376
	6	GGTGGGTTACTGACACCA<br>CTAACGTGTACCACCCGT	1527	6	CTGGGCCTGGTGCTAGTCA<br>CCTGTTACCTCGATCCTAC	1100
	7	TGCAGACATCCCAGAGGT<br>CCTGTTACCTCGATCCTAC	878			
LJK2-2 (*MAstV–24*)	1	ATGGATCAGTTGTTTCGC<br>AGTATCATCTGGTTGCCA	999	1	ATGGATCAGGTGTTTCGTCG<br>GAGACCTCAACGGATGGG	1397
	2	TCCCTGTTTGATGCACTTGG<br>ATCCTCACGTGTGAGACGT	837	2	ACCACTCGTGTTGTGTCATTG<br>ATCACATTGCTGCAACGC	668
	3	GATTCTGAGAATGGCTCATCT<br>GTATCAACCTTAATGGGTGG	1060	3	GGTCAACGTCTCACACGTGA<br>GGGCATACTGGAACTTCTC	1034
	4	CGCTTCCTTGTGCACCCT<br>GAGGAGGTTATGGACATACC	822	4	GCATCCTGACTTTCCAGTCA<br>CGCAGAAGCTGTTGTGGTA	716
	5	GGTTGACTGGACTCGTTATG<br>CTGTTGTAGTCTTAGTGGTAGG	870	5	GGTCAAGGAACGTACTGAAG<br>CCATTTGTGGACACTGTGGT	916
	6	GAAATGATGGCCAATCAG<br>AGCCAAAGTTCACCAGACCA	740	6	CAGCGTAACAGGAGAACCC<br>GATTCATATTCCAATTGGTG	772
	7	TGCTGGGCCAACCATAGAG<br>GAGCATCAACACCCATGGG	869	7	CTGCTGGGCCAACCATAG<br>CCCGCCTGTAAGGCAGAT	719
	8	CAGCCTCATCTGTCTTTGG<br>GAGCTGTACCCTCGATCCT	950	8	CTCAGACCTCTGGGCGCT<br>GAGCTGTTACCTCGATCCT	1011

[a]primers used for amplification

[b]primers used for verification

Table S3. The Information regarding the reference sequences and 28 obtained sequences in this study

Strain	Genbank	Strain	Genbank
Astrovirus_dogfaeces/Italy/2005	FM213330	Human_astrovirus_7	Y08632
Astrovirus_MLB1	FJ222451	Human_astrovirus_8	AF260508
Astrovirus_MLB2	JF742759	Human_astrovirus_VA1/HMO-C	GQ415662
Astrovirus_rabbit/Nausica/2008/ITA	JN052023	Human_astrovirus_VA2/HMO-A	GQ415660
Astrovirus_rat/RS118/HKG/2007	HM450381	Human_astrovirus_VA3/HMO-B	GQ415661
Astrovirus_swine/PoAstV12-4/Canada/2006	HM756259	MiAstV_SMS	GU985458
Astrovirus_swine/PoAstV14-4/Canada/2006	HM756260	Mink_astrovirus	AY179509
Astrovirus_swine/PoAstV16-2/Canada/2006	HM756261	Mouse_astrovirus_M-52/USA/2008	JF755422
Avian_nephritis_virus_1	AB033998	MOxAstV_CH18	MK211323
Bat_astrovirus	EU847144	OvAstV_CH16	KY859988
Bat_astrovirus	EU847145	OvAstV_CH17	MK286562
Bat_astrovirus	EU847155	OvAstV_S5.1	MK404648
Bat_astrovirus_Hp/Guangxi/LC03/2007	FJ571074	OvAstV_S6.1	MK404649
Bat_astrovirus_Tm/Guangxi/LD38/2007	FJ571065	OvAstV_UK/2013/ewe/lib01454	LT706531
Bat_astrovirus_Tm/Guangxi/LD71/2007	FJ571067	OvAstV_UK/2014/lamb/lib01455	LT706530
Bat_astrovirus_Tm/Guangxi/LD77/2007	FJ571066	OvAstV-1	NC_002469
BAstV-GX27/CHN/2014	KJ620980	OvAstV-2/Hungary/2009	JN592482
BoAstV/CHN/HLJ-2/2019	MW373714	PAstV5/33/USA	JF713711
BoAstV/JPN/Hokkaido11-55/2009	LC047790	PAstV5/AstV-LL-2/China/2006	KP747574
BoAstV/JPN/Hokkaido12-18/2009	LC047792	PAstV5-AH29-2014	MT642595
BoAstV/JPN/Hokkaido12-25/2009	LC047793	PAstV5-US-IA122	JX556693
BoAstV/JPN/Kagoshima1-2/2014	LC047795	PoAstV-3_NI-Brain/9-2016a/HUN	KY073229
BoAstV/JPN/Kagoshima2-3-2/2015	LC047798	PoAstV-3_USA/IA/7023/2017	KY940545
BoAstV/JPN/Kagoshima2-38/2015	LC047800	PoAstV5/JPN/HgTa2-1-3/2015	LC201615
BoAstV_BH89/14	LN879482	PoAstV5/JPN/MoI2-3-1/2015	LC201618
BoAstV_CH13/NeuroS1	KX266902	PoAstV5/Swine/CHI/FB032/2017	MZ819172
BoAstV_CH13/NeuroS1	KX266905	PoAstV5/Swine/CHI/FB033/2017	MZ819171
BoAstV_CH13/NeuroS1	KX266906	PoAstV-5-LL-2	KP747574
BoAstV_CH13/NeuroS1	KX266908	Porcine_astrovirus_1	AB037272
BoAstV_CH13	NC_024498	Porcine_astrovirus_4	JF713713
BoAstV_CH15	KT956903	Porcine_astrovirus_PAstV-2/2007/HUN	GU562296
BoAstV_KagoshimaSR28-462	LC341267	Sichuan_takin_astrovirus_LLT03	NC_037655
BoAstV_NeuroS1	KF233994	SWUN/1-FCFX3/2020/Goat	OM890921
BoAstV_VC34/346	MK987100	SWUN/1-LFX2/2020/Goat	OM890915
BoAstV_VC65/698	MK987103	SWUN/1-LJK3/2020/Goat	OM890932
BoAstV-Neuro-Uy	MK386569	SWUN/2-FCFX3/2020Goat	OM890917
Bottlenose_dolphin_astrovirus_1	FJ890355	SWUN/2-LFX2/2020/Goat	OM890911
Bovine_astrovirus_B34/HK	HQ916315	SWUN/2-LJK3/2020/Goat	OM890923
Bovine_astrovirus_B76/HK	HQ916316	SWUN/3-10/2020/Goat	OM890910
Bovine_astrovirus_B76-2/HK	NC_023630	SWUN/ACFX6/2021	OK107515
BufAstV/CN/NNA14	MT499772	SWUN/CCFX2/2020/Goat	OM890919
BufAstV/CN/NNDs2	MT521688	SWUN/ECJK3/2021	OK107514

California_sea_lion_astrovirus_1	FJ890351	SWUN/F1/2019/Goat	OK107512
California_sea_lion_astrovirus_2	FJ890352	SWUN/F2/2019/Goat	OK107513
CapAstV_G2.1	MK404645	SWUN/FCFX1/2020	OM890920
CapAstV_G3.1	MK404646	SWUN/FCJK2/2020	OM890922
CapAstV_G5.1	MK404647	SWUN/HJK3/2020	OM890925
CapAstV_SWUN/F4/2019	MZ005893	SWUN/JCFX1/2020	OM890927
CcAstV/roe_deer/SLO/D12-14/2014	MN150125	SWUN/JCFX3/2020	OM890914
CcAstV/roe_deer/SLO/D5-14/2014	MN150124	SWUN/JCFX4/2020	OM890926
Duck_astrovirus_1	FJ434664	SWUN/JCFX5/2020	OM890929
Feline_astrovirus	AF056197	SWUN/JCFX6/2020	OM890912
HuAstV_VA1/HMO-C	KJ920196	SWUN/JCJK1/2020	OM890913
HuAstV_UK1	KM358468	SWUN/JCJK3/2020	OM890928
HuAstV-SG	GQ891990	SWUN/JK13/2020	OM890918
Human_astrovirus_1	L23513	SWUN/JK3/2020	OM890930
Human_astrovirus_2	L06802	SWUN/JK4/2020	OM890909
Human_astrovirus_3	AF117209	SWUN/LFX1/2020	OM890916
Human_astrovirus_4	DQ070852	SWUN/LJK2-1/2020	OM890924
Human_astrovirus_5	AB037274	SWUN/LJK2-2/2020	ON571622
Human_astrovirus_6	Z46658	Turkey astrovirus TAstV/CO/01	EU143845
